# Experimental and Ab Initio Characterization of Mononuclear Molybdenum Dithiocarbamates in Lubricant Mixtures

**DOI:** 10.1021/acs.langmuir.1c00029

**Published:** 2021-04-13

**Authors:** Gabriele Losi, Stefan Peeters, Franck Delayens, Hervé Vezin, Sophie Loehlé, Benoit Thiebaut, Maria Clelia Righi

**Affiliations:** † Department of Physics and Astronomy, University of Bologna, 40127 Bologna, Italy; ‡ Total Marketing and Services, Chemin du Canal BP 22, 69360 Solaize, France; § University of Lille, CNRS, UMR 8516 - LASIRE - Laboratoire Avancé de Spectroscopie pour les Interactions, la Réactivité et l’Environnement, F-59000 Lille, France; ∥ Department of Physics, Informatics and Mathematics, University of Modena and Reggio Emilia, I-41125 Modena, Italy

## Abstract

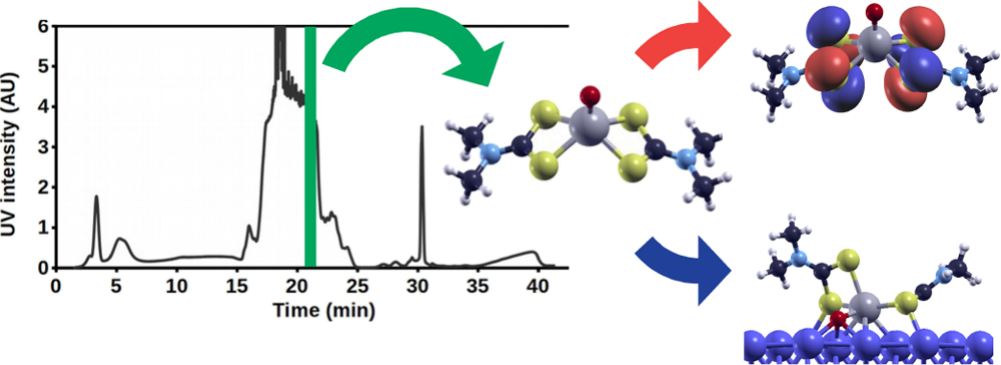

Molybdenum dithiocarbamates (MoDTCs) are a class of lubricant additives widely employed in automotives. Most of the studies concerning MoDTC take into account the dimeric structures because of their industrial relevance, with the mononuclear compounds usually neglected, because isolating and characterizing subgroups of MoDTC molecules are generally difficult. However, the byproducts of the synthesis of MoDTC can impact the friction reduction performance at metallic interfaces, and the effect of mononuclear MoDTC (mMoDTC) compounds in the lubrication has not been considered yet in the literature. In this study, we consider for the first time the impurities of MoDTC consisting of mononuclear compounds and combine experimental and computational techniques to elucidate the interaction of these impurities with binuclear MoDTC in commercial formulations. We present a preliminary strategy to separate a commercial MoDTC product in chemically different fractions. These fractions present different tribological behaviors depending on the relative amount of mononuclear and binuclear complexes. The calculations indicate that the dissociation mechanism of mMoDTC is similar to the one observed for the dimeric structures. However, the different chemical properties of mMoDTC impact the kinetics for the formation of the beneficial molybdenum disulfide (MoS_2_) layers, as shown by the tribological experiments. These results help to understand the functionality of MoDTC lubricant additives, providing new insights into the complex synergy between the different chemical structures.

## Introduction

Friction is an unavoidable phenomenon which results in massive economic and environmental costs.^1^ The search for innovative solutions in tribology pushes the scientific community to design lubricant additives which are more efficient in reducing friction than the currently employed ones while limiting their negative effects for the environment by decreasing their concentration in the formulations. This difficult task requires a complete understanding of the mechanism of action of the currently employed lubricant additives, which are often involved in very complex reactivity.^2,3^

Molybdenum dithiocarbamates (MoDTCs) are a family of different chemical compounds widely used as friction modifiers in the automotive industry. Organo-moybdenum compounds, in fact, are among the most successful lubricant additives in the boundary lubrication regime, where the asperity contact between steel surfaces generates extreme conditions in the tribological systems.^4−6^ At these contacts, MoDTC breaks down due to the interaction with the substrate and the high local temperatures and pressures. The molecular fragments resulting from the dissociation can be reorganized into molybdenum disulfide (MoS_2_), a remarkable solid lubricant, under the effect of the mechanical stresses. MoS_2_ is a layered material, and its mechanism of function consists in weak interlayer forces that offer very small resistance to sliding, ensuring protection for the metallic surfaces in relative motion.^7^

The synergistic interaction of MoDTC structures with ZnDTP, common antiwear additives, is well established in the literature.^8−10^ In fact, MoDTC can easily oxidize when exposed to air. The new oxygen atoms can substitute the sulfur atoms present in the molecules in different positions, especially in the ligand position,^11,12^ leading to a wide variety of similar chemical structures. However, oxidation is not the only source of complexity in this family of compounds as structures with one or three Mo atoms have also been reported.^13^ While most of the scientific literature focuses on the dimeric structures of MoDTC, mononuclear structures can also play a role in commercial formulations. These alternative structures of MoDTC could be the result of chemical equilibria inside the mixture, and it is highly desirable to better understand the origin and the mechanism of action of these compounds. To this day, the description of mononuclear MoDTC (mMoDTC) structures appears only in few patents,^14,15^ a study by Zaimovskaya et al.,^16^ and a recent publication by Al Kharboutly et al., in which the authors describe a successful synthesis of a mononuclear compound able to outperform dimeric structures at high pressures.^17^

In this study, we combined experimental techniques and ab initio simulations to investigate the role of different MoDTC structures in the lubricant mixtures, with a particular focus on the properties of mMoDTC. First, we separated a sample of a commercial product containing MoDTC by means of flash chromatography into chemically different fractions. By characterizing the most relevant fractions, we obtained evidence on the presence of mMoDTC in the samples. Tribological experiments were performed to investigate the friction reduction performance of the different fractions compared to the commercial product. For a systematic description of the chemical properties of the mononuclear compounds, we carried out static density functional theory (DFT) calculations and we evaluated for the first time geometric and electronic properties of mMoDTC, estimated the strength of its bonds, and studied its interaction with metallic substrates and its possible decomposition mechanism on iron. We also evaluated the reaction energy of a simplified chemical equilibrium between mononuclear and binuclear structures. All the results obtained for the mononuclear compounds are compared to the previous observations derived for the binuclear compounds in an attempt to shed light on the intricate functionality of these lubricant additives.

## Methods

### Experimental Techniques

As a commercial product, MoDTC is available in mixtures where similar chemical structures can be present at the same time. MoDTC molecules may have different alkyl chain lengths, degrees of oxidation, and numbers of Mo atoms.^11,13^ Sakuralube 525 (S525) was chosen for this work because of its industrial relevance. It consists of a mixture of MoDTC structures, dissolved in base oil, with 8- and 13-membered lateral chains in their dithiocarbamate units. We performed a separation of S525 by means of flash chromatography. A sample containing 1 g of S525 in 4 mL of cyclohexane was injected on the head of a GRACE Reveleris HP Silica 20 μm cartridge, previously conditioned with cyclohexane. A VWR International LaFlash chromatograph, coupled with a Gilson FC204 fraction collector, was employed for the separation. The eluted species were detected by measuring their ultraviolet (UV) absorption at 254 nm. Table 1 summarizes the program of the separation, where the eluting solvents A, B, C, and D correspond to cyclohexane, dichloromethane, ethyl acetate, and 2-propanol, respectively. The flow rate was 20 mL/min for the whole duration of the separation. The fractions were collected from minute 10 of the run, and each vial contained 25 mL of the liquid. X-ray fluorescence (XRF) and mass spectrometry revealed that the seventh, eighth, and ninth collected fractions contained the highest amount of MoDTC compounds, as shown in the Supporting Information. For the subsequent characterization, the solvent of these fractions was evaporated overnight under a flux of nitrogen. A portion of the solid obtained was dissolved in isooctane to record electronic spectra on a Varian Cary 300 BIO UV/visible spectrophotometer, while the remaining sample was dissolved in mineral group III oil with a kinematic viscosity of 4 cSt at 100 °C for the tribological tests.

**Table 1:** Program for the Separation of S525 with Flash Chromatography^a^

step	time (min)	% A	% B	% C	% D
1	0 → 10	100	0	0	0
2	10 → 25	100 → 0	0 → 100	0	0
3	25 → 33	0	100 → 50	0 → 50	0
4	33 → 45	0	0	0	100

a The arrows in the table indicate the initial and final times and compositions of the eluting solvent. Steps 1 and 4 are isocratic.

To better understand the chemical composition of the separated fractions, we performed mass spectrometry and electron paramagnetic resonance (EPR) spectroscopy. Mass spectrometry experiments were carried out using a hybrid quadrupole-ion mobility-time-of-flight mass spectrometer (Synapt G2Si, Waters, Manchester, UK) equipped with an electrospray (ESI) source. The ESI source was operated in the positive ionization mode, and the parameters were set as follows: a sampling cone of 80 V, a capillary of 3 kV, a source offset of 80 V, a source temperature of 100 °C, a desolvatation temperature of 250 °C, and a nitrogen gas flow of 500 L/h. Experiments were accomplished in the *V* resolution mode in the 50–2000 *m*/*z* range. Data acquisition and data treatment were performed using the MassLynx (version 4.1) software.

The echo field sweep EPR spectra of an equivalent set of fractions were recorded by using a Bruker ELEXYS 580-FT spectrometer operating at the X and Q bands. A π/2−τ–π/2 echo sequence was used with pulse lengths for π/2 = 16 ns and π = 32 ns. The hyperfine sublevel correlation spectroscopy (2D-HYSCORE) spectra were recorded with the following pulse scheme: π/2−τ–π/2–*t*_1_–π–*t*_2_–π/2–*t* echo and a four-step phase cycling where the echo is measured as a function of *t*_1_ and *t*_2_, with *t*_1_ and *t*_2_ being incremented by steps of 16 ns from their initial values. All the measurements were carried out at 20 and 5 K using a CoolEdge cryofree cryostat system.

The tribological tests were carried out on polished AISI 52100 hardened steel discs by employing fractions 7, 8, and 9 as lubricants, with a molybdenum concentration of 50 ppm. S525 dissolved in the same group III mineral oil, with the same molybdenum concentration as in the fractions, was chosen as a reference lubricant. All the lubricants were deposited directly on the discs immediately before the run. An Anton Paar MCR302 Rheometer equipped with a tribometer assembly (T-PID/44 three balls on the disk measurement cell) was used. The rotation speed increased from 4.7 to 377 mm/s over 750 s, with a total test duration of 20 min. The temperature of the system was 110 °C, and the contact pressure was 700 MPa.

### Computational Techniques

**Figure 1:**
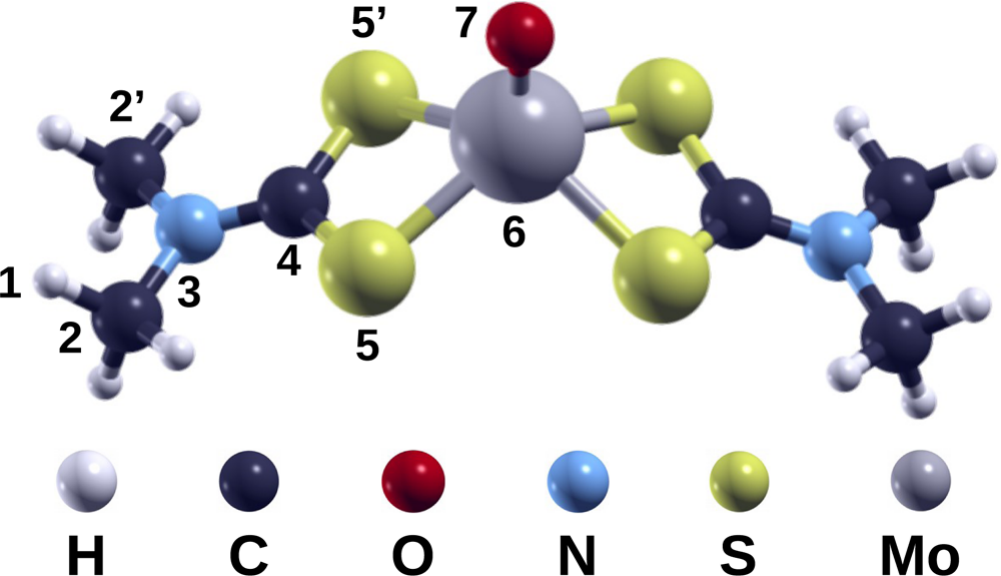
Molecular structure of mMoDTC.

We considered the mMoDTC structure shown in Figure 1 for our computational study. In this structure, a single Mo atom is coordinated by an oxide and two dithiocarbamate units. We considered methyl groups as lateral chains in the dithiocarbamate units, which are shorter than the ones in the experimental samples, to alleviate the computational cost. Such an approximation was already justified in our previous publications regarding binuclear MoDTC compounds and organophosphorus additives.^12,18−20^ In several publications, mMoDTC contains two oxide ligands and the Mo atom has an oxidation number of +6.^14−16^ In particular, Al Kharboutly et al. specifically synthesized the mononuclear compound with two oxygen atoms.^17^ The chemical structure we considered for our computational study differs from the ones already proposed in the literature because of the results provided by mass spectrometry and EPR, as explained in detail in the next section.

We characterized the properties of mMoDTC by means of static calculations in the DFT framework implemented in the Quantum ESPRESSO package.^21^ The DFT calculations were carried out by following the pseudopotential/plane wave approach, with periodic boundary conditions applied to the supercells. The Perdew–Burke–Ernzerhof (PBE) functional^22^ was adopted to describe electronic exchange and correlation. To evaluate the properties of isolated mMoDTC and its fragmentation energy, we decided to truncate the plane wave expansion by selecting cutoffs of 40 and 320 Ry for the wave functions and for the charge density, respectively, as the pseudopotentials employed in this work were ultrasoft. For all the calculations, the integrations of the charge density were carried out at the gamma point. Spin polarization was taken into account in order to allow the spin multiplicity of the different chemical species to vary. A Gaussian smearing of 0.02 Ry was added to better describe the occupations of the electronic states around the Fermi level. The geometry optimizations were stopped when the total energies and the forces on the atoms converged below the thresholds of 1 × 10^–4^ Ry and 1 × 10^–3^ Ry/bohr, respectively.

For part of the calculations on the fragmentation of isolated mMoDTC, the Gaussian 09 software^23^ was used. The basis set used for these calculations was def2-TZVP,^24,25^ and all the computational parameters were already described in a previous publication.^12^

For the isolated chemical structures, a cubic supercell with an edge of 60 bohr units was chosen to avoid interactions with the periodic replicas. The Avogadro 1.2.0 software^26,27^ was employed to generate an initial guess for the geometry of the chemical structure, which was then optimized without imposing any symmetry constraint.

To simulate the interactions of the lubricant with a metallic substrate, Fe(110) was considered as it is the most favorable surface of this metal.^28,29^ For the calculations including the iron slabs, the cutoffs for the kinetic energy of the wave functions and for the charge density were lowered to 30 and 240 Ry, respectively, for the reasons explained in our previous work.^30^ Static calculations with mMoDTC and its fragments on the iron slabs were carried out in  supercells in the units of the lattice parameter of bulk iron. In this case, the iron slabs were composed of four atomic layers.

## Results

### Separation of the Additive Mixture and Characterization of the Collected Fractions

**Figure 2:**
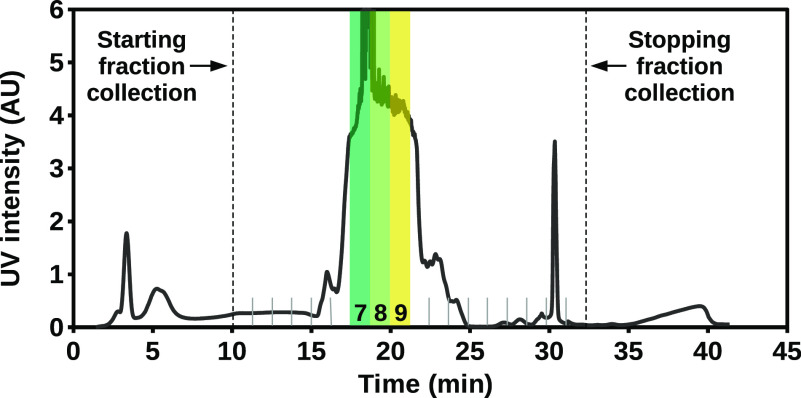
Chromatogram of the flash separation of S525. The collected fractions are marked on the bottom of the plot, with fractions 7, 8, and 9 highlighted with their corresponding colors.

Figure 2 shows the chromatogram of the separation of the S525 mixture. The products obtained during the run can be grouped in four parts, ordered by increasing polarity, which were eluted in the respective steps of the run

1. Before 10 min: base oil.
2. Between 15 and 25 min: the majority of MoDTC compounds.
3. Between 25 and 33 min: few polar MoDTC compounds.
4. After 35 min: degradation products of MoDTC.

Infrared (IR) spectra confirmed the absence of MoDTC compounds in the fractions eluted before 10 min and their presence in the peaks between 15 and 33 min of the run. The IR band belonging to the diagnostic Mo=O vibration between 900 and 1000 cm^–1^^6,12^ was also detected in the last fraction after 35 min. However, due to the large amount of impurities in the last fraction and the complete lack of MoDTC compounds in the first fractions, we collected only the fractions belonging to the two central groups of peaks, between 10 and 33 min, discarding the rest of the elution. The collected fractions contained mixtures of several MoDTC compounds which were difficult to purify with such a protocol due to their very similar chemistry. Among the fractions collected from the main group between 15 and 25 min, we focused our attention on fractions 7, 8, and 9 as they were the most concentrated ones and they showcased a color transition from green to yellow, as indicated in Figure 2. Electronic spectra of these fractions are included in the Supporting Information.

### Characterization and the Tribological Test of the Collected Fractions

**Figure 3:**
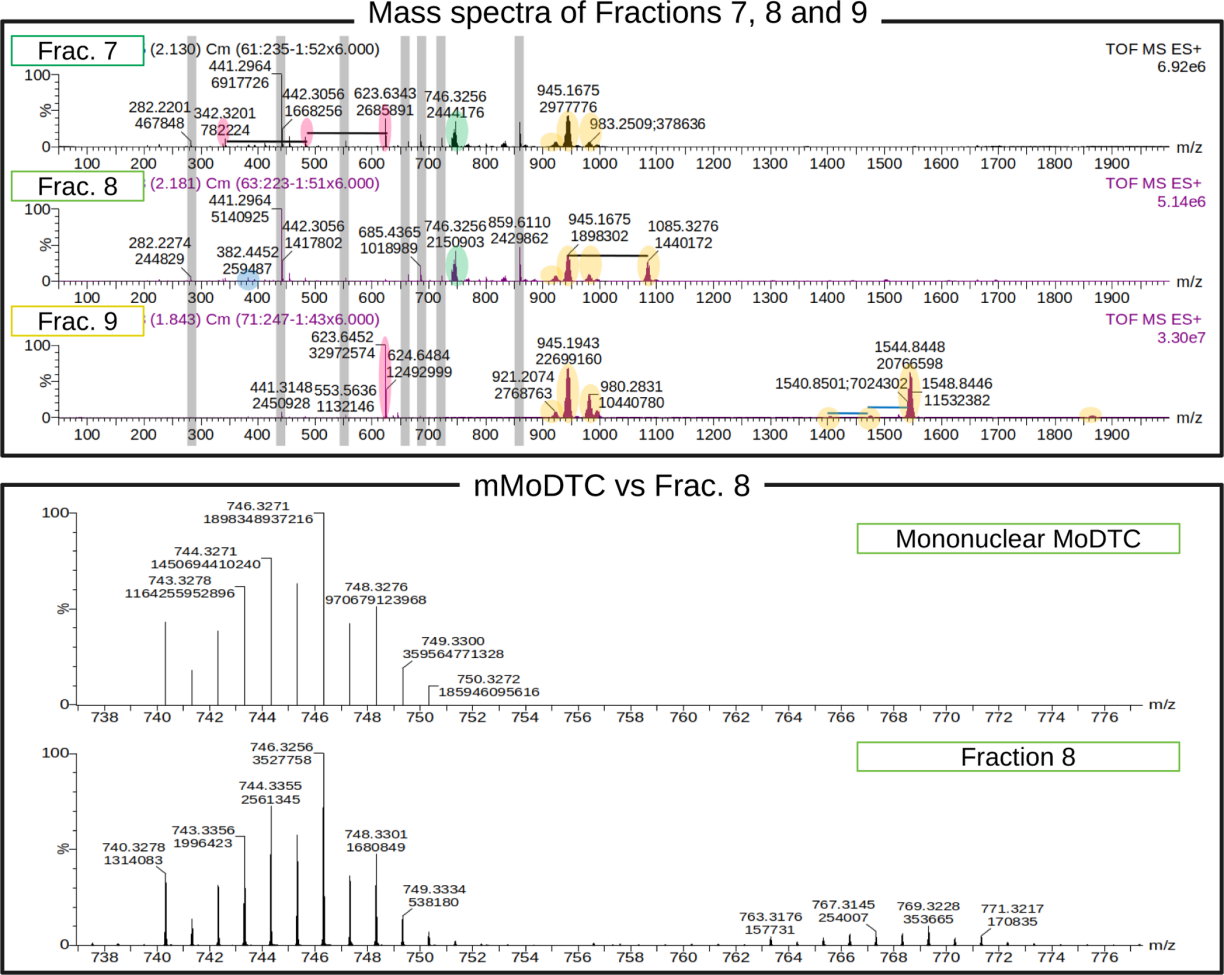
Top panel: mass spectra of fractions 7, 8, and 9. Different families of the compound are grouped by color. Bottom panel: comparison between the theoretical spectrum of mMoDTC and the corresponding region in the spectrum of fraction 8.

As shown in Figure 3, mass spectra indicate the presence of three groups of compounds in these fractions: mMoDTC at around 746.3 *m*/*z* (green overlay), binuclear MoDTC at around 924–945 *m*/*z* (orange overlay), and impurities, such as primary and secondary amines, dialkyl thiourea, and ionic compounds originating by the clustering of two or more MoDTC complexes. In particular, the ratio between mononuclear and binuclear MoDTC varies among the different collected fractions. The approximate mononuclear/binuclear ratios in fractions 7, 8, and 9 are 0.26, 0.5, and 0, respectively, implying that the richest fraction in terms of mMoDTC is fraction 8. The separation protocol could be further optimized to obtain even better separations of the different MoDTC compounds. However, flash chromatography allowed us to collect chemically different MoDTC samples already after a single run. The presence of aggregates of several MoDTC molecules can be explained by considering the ionization technique used in this work. Electrospray ionization (ESI) is a soft ionization technique, implying that the dissociation and the transformation of the compounds are minimized. On the other hand, this technique favors the formation of adducts. Proton or sodium adducts can often be observed depending on the surrounding molecules, especially for binuclear MoDTC molecules, and the aggregation of several ionized species may occur as well. While the sodium adduct is the predominant form of binuclear MoDTC observed with ESI, the sodium adducts of the mMoDTC appear only as a minority and the proton adducts are undetected. The predominant form of mMoDTC in these samples corresponds to the chemical formula MoOS_4_N_2_C_34_H_68_. Such a compound is similar to the chemical structure shown in Figure 1 and showcases eight-membered carbon chains instead of methyl groups in their dithiocarbamate units. We believe that this special form of mMoDTC is a radical cation, with a Mo(V) in its core, because its signal is much stronger than its corresponding sodium adduct, while this is most often not the case with binuclear compounds. For binuclear compounds, the signal of the sodium adduct is stronger than the one at lower *m*/*z*. One might suggest that a radical cation with Mo(V) would be the result of the removal of one electron from a neutral compound with the same stoichiometry and Mo(IV). However, this is unlikely because the sodium adducts of the mononuclear compounds are the minority in the mixture, while the proton adducts and larger aggregates are not observed in these mass spectra, as opposed to the binuclear compounds. Therefore, we believe that in the lubricant mixtures, two forms of mMoDTC coexist: one radical cation with Mo(V), which is predominant, and a neutral compound with Mo(IV).

**Figure 4:**
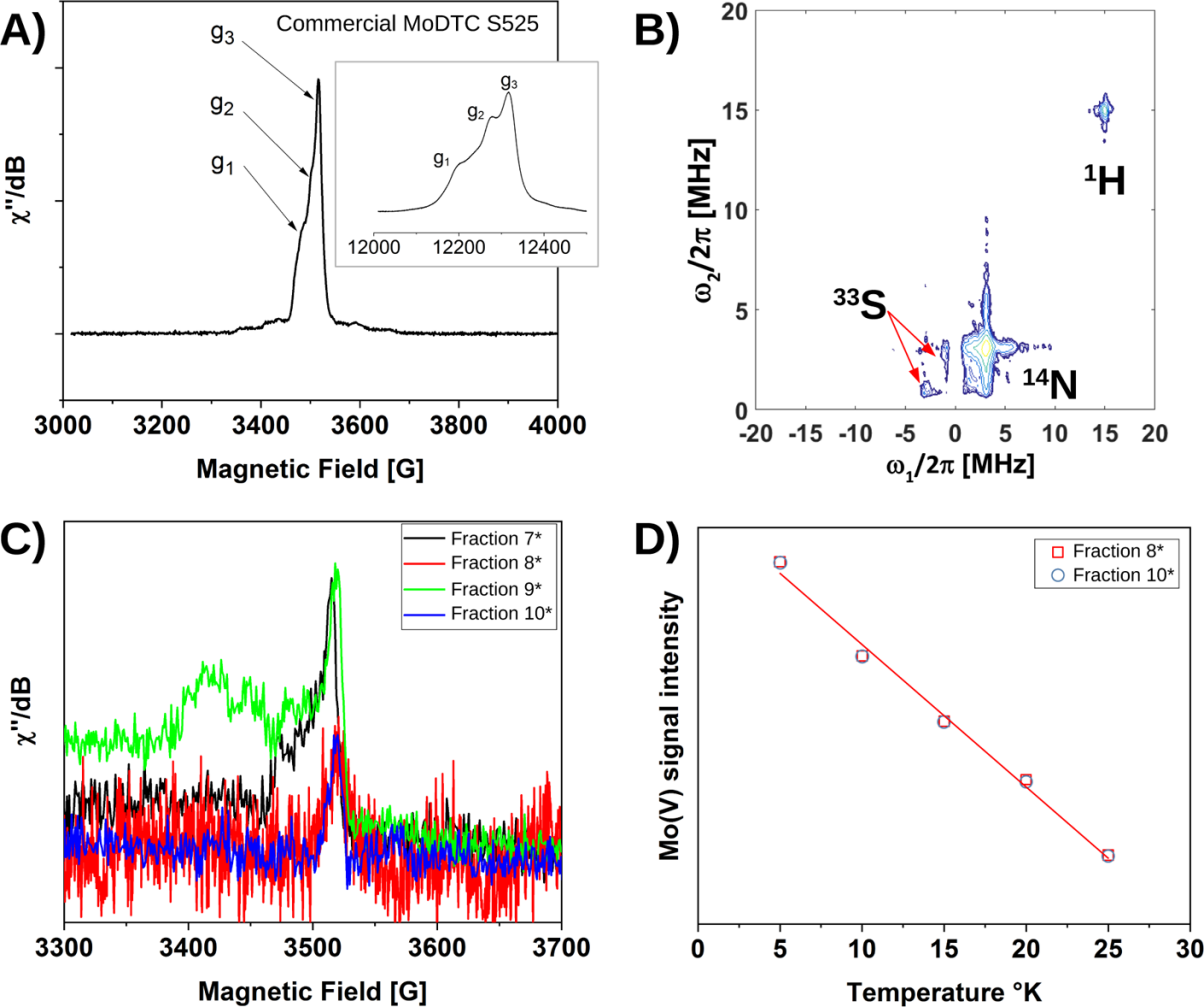
EPR spectra of the MoDTC samples. (A) Echo field sweep EPR spectrum of the commercial S525 mixture measured at the X-band and at 20 K, with the Q-band echo spectrum at 20 K as an inset. (B) 2D-HYSCORE spectrum at 20 K of the commercial S525 mixture. (C) Echo field sweep EPR spectrum of the separated fractions measured at the X-band and at 5 K. (D) Intensity of the Mo(V) signal vs the temperature, indicating the Curie behavior and, therefore, the paramagnetic nature of the species in fractions 8* and 10*.

To better understand the electronic properties of the compounds identified by mass spectrometry, we performed EPR spectroscopy on the commercial mixture of MoDTC compounds and on a set of equivalent fractions from a different separation. We will mark the fractions obtained by the new separation, in which we followed the same experimental procedure as described above, with an asterisk in the following. Panel A of Figure 4 shows the echo field sweep spectrum of commercial MoDTC recorded at 20 K. This signal is typical of the Mo(V) d^1^ electron spin state.^31,32^ The X-band spectrum shows that the Mo(V) complex is on an octahedral symmetry, as indicated by the anisotropy of the g-factor with three eigenvalues. To better visualize the g-anisotropy, a Q-band spectrum has been recorded (inset). The measured values of *g*_1_, *g*_2_, and *g*_3_ are 1.99, 1.98, and 1.97, respectively. To gain more insight into the nuclear environment of the Mo(V) coordination shell, a 2D-HYSCORE experiment was performed, as shown in Panel B. In the (−,+) quadrant, two antidiagonal cross peaks that split at 2.3 MHz are present. They are the result from a coupling with ^33^S atoms with an isotropic hyperfine interaction *a*_iso_ of 4.2 MHz. In the (+,+) quadrant, ^14^N coupling is measured with a strong dipolar interaction. Weak coupling of the proton is also observed. The fractions analyzed by flash chromatography were also measured by echo field sweep detection. The results shown in panel C indicate that all the fractions contain Mo(V). Fractions 8* and 10* show an isotropic signal with a g-factor of 1.97. Measuring this signal as a function of temperature in the range of 5–25 K provides a Curie behavior (panel D), indicating that the complexes in fractions 8* and 10* are paramagnetic and hence monomeric. Fractions 7* and 9* showcase an additional feature around *g* = 2.03. This signal is typical of S radical species which are formed by a progressive bond breaking of the sulfur atom in the coordination shell of Mo(V).

The combination of mass spectrometry and EPR spectroscopy supports the hypothesis of the presence of a mononuclear structure with a Mo(V) atom and the molecular formula MoOS_4_N_2_C_34_H_68_. The Curie behavior observed in fractions 8* and 10* indicates that the oxidation number of +5 belongs to a paramagnetic chemical structure, namely, the mononuclear compound. Indeed, the dimeric compound is antiferromagnetic. A structure compatible with the observations from mass and EPR spectra is the radical cation mononuclear complex described above. Possible counterions could be dialkyl dithiocarbamates (DTCs) or their dimers, called bis-DTCs, although complex ionic structures based on MoDTC have not been described yet in the literature. Neutral mononuclear compounds, with the oxidation number of +4 on the Mo atom, can be present as well in the fractions, yet they cannot be detected by EPR because their electronic structures are closed subshells. Therefore, in the following, we focused our attention on both the cationic and neutral forms of mMoDTC.

**Figure 5:**
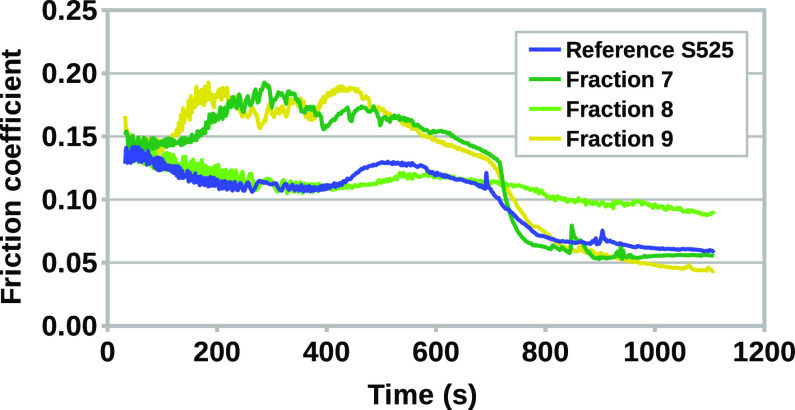
Tribological test of the considered fractions and the reference mixture.

Figure 5 shows the results of the tribological tests where fractions 7, 8, and 9 and a reference mixture of S525 were employed as lubricants. Overall, the best tribological performance was provided by the full mixture of MoDTC structures. In the case of the reference lubricant, in fact, the friction coefficient remains at the lowest levels both before and after the tribochemical activation of the additives to form MoS_2_, which occurs around 700 s. Fractions 7 and 9, which contain the least amount of mononuclear structures, provide an initial increase in friction, probably caused by high severity at the contact. However, the two systems are able to eventually reach low friction, with values comparable to the ones observed with S525. Surprisingly, fraction 8, which contains the highest amount of mononuclear structures, initially follows the same profile as the reference lubricant, although the activation of the friction reduction additives does not occur during the test. It should be noted that a variety of MoDTC structures are present at the same time in all the considered fractions. However, these compounds appear to play a synergistic role when they are combined into S525 as the different structures have specific impacts on the tribological behavior of the lubricant, while their friction reduction performance is limited when the original mixture is fractioned.

To better understand the role of mMoDTC in lubricant mixtures, compared to binuclear structures, we focused our computational investigation on the cationic and neutral forms of the mononuclear structure indicated in Figure 1.

### Calculated Molecular Properties

We started our computational study by calculating the ionization energy *E*_I_ of mMoDTC and compared it to the corresponding energy of the dimeric structure of MoDTC, which we called standard MoDTC (sMoDTC) in our previous publications. The calculated values for *E*_I_, simply obtained by subtracting the total energies of the neutral complexes from the structures with one missing electron, turned out to be 5.3 and 6.2 eV for mMoDTC and sMoDTC, respectively, making the mononuclear compounds easier to ionize than the binuclear compounds.

**Figure 6:**
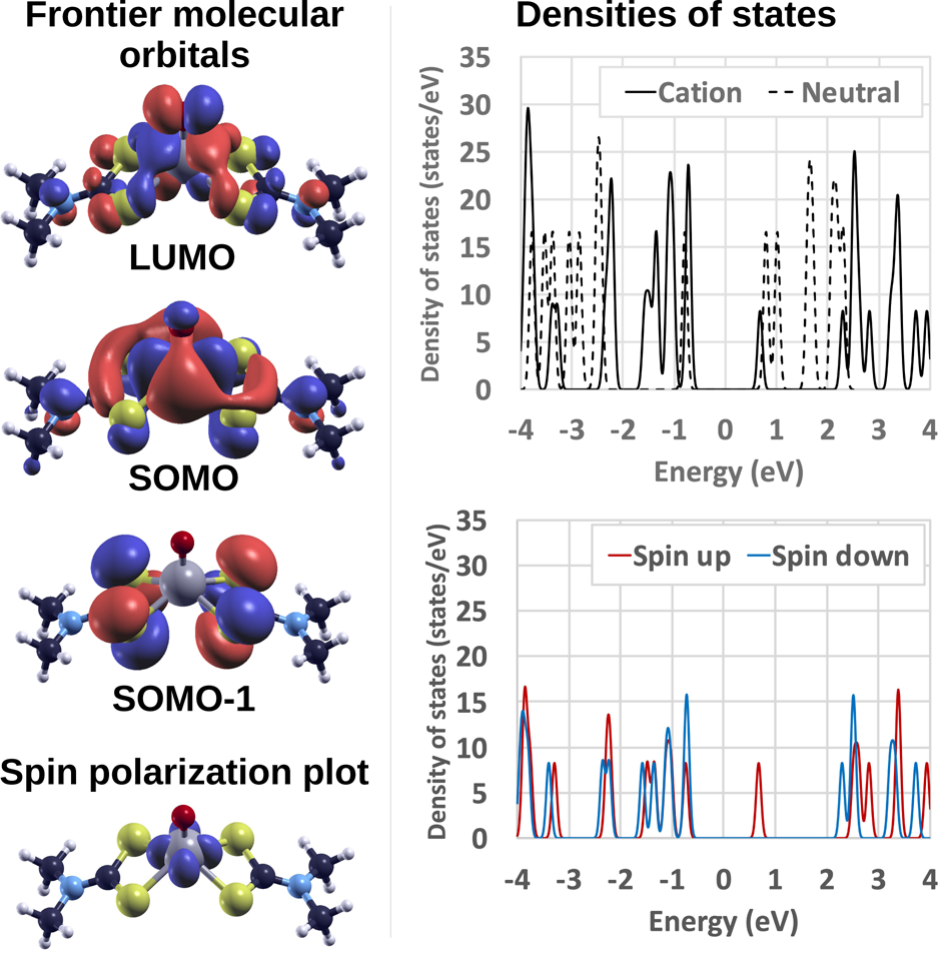
Electronic properties of mMoDTC. Top left: frontier molecular orbitals of the mMoDTC cation. The isovalue for the LUMO and SOMO-1 is 0.002, while for the SOMO, the isovalue is 0.0005 to better visualize the details of the plot. The red and blue colors of the isosurfaces represent the positive and negative signs, respectively, of the electronic wave function. Bottom left: spin polarization plot, where the blue coloring of the isosurface around the Mo atom indicates an excess of negative spin. The isovalue for this plot is 0.01. Top right: DOSes for cationic (solid line) and neutral (dashed line) mMoDTC. Bottom right: densities of spin-up (red) and spin-down (blue) states in cationic mMoDTC. The DOSes were shifted in order to set the Fermi energy to 0 in all the plots.

The cationic form of mMoDTC is a radical as a single non-bonding electron is left on the Mo atom. Such an unpaired electron is hosted in a singly occupied molecular orbital (SOMO), shown in the top-left panel of Figure 6, along with the highest fully occupied molecular orbital (SOMO-1) and the lowest unoccupied molecular orbital (LUMO). The SOMO is the result of a combination of atomic orbitals in which three 4d orbitals of molybdenum account for more than 70% of the molecular wave function. All the contributions above 5% determining SOMO-1 come from 3p orbitals of the S atoms, while more than 65% of the LUMO is determined by one 2p orbital of oxygen and two 4d orbitals of molybdenum. The shapes of the frontier molecular orbitals of the cationic and neutral forms of mMoDTC are compared in the Supporting Information, and a good match can be observed for the populated orbitals of the two species. The shape of the SOMO of the cation, in combination with the spin polarization plot shown in the bottom-left panel of Figure 6, reveals that the unpaired electron, characterized by a negative spin, is mainly localized on the Mo atom. The densities of states (DOSes) of the cationic and neutral forms of mMoDTC, shown in the top-right panel of Figure 6, differ because the missing electron in the cation induces a slight rearrangement of the nuclei, which, in turn, leads to an energy shift and a change in the density of the electronic states. The SOMO–LUMO gap calculated for the cation is approximately 1.42 eV, while for the neutral complex, the HOMO–LUMO gap is approximately 1.59 eV, compared to 2.72 eV, as in the case of sMoDTC. However, the calculated values for the gap energies are merely representative due to the well-known fundamental gap problem in DFT.^33^ To better visualize the asymmetry in the DOSes occupied by electrons with different spins, the DOS of cationic mMoDTC can be decomposed in the contributions of the spin-up and spin-down electrons, as shown in the bottom-right panel of Figure 6, which demonstrates the increased amount of occupied states for electrons with spin down in proximity of the Fermi energy.

To summarize, the monomer is easier to ionize than the dimer. It is possible that mMoDTC in the mixtures is originally present as both neutral and cationic compounds. However, under tribological conditions, electron transfer among chemical species could occur because of the high energies generated by mechanical stresses acting on the system. When cationic mMoDTC is formed, it can quickly react with other species present in the mixture because of the radical electron localized on the central metallic unit. The different electronic properties of mMoDTC and its capability to bind to other MoDTC structures can explain the different color of the samples containing the monomer with respect to samples containing only the dimeric structures.

The bond lengths and angles of mMoDTC obtained from geometry optimization turned out to be similar to the corresponding values in sMoDTC, with the discrepancies in the bonds and the angles laying below 2 and 7% in absolute value, respectively. The complete analysis on the structural data is reported in Table 2.

**Table 2:** Lengths and Angles of Selected Bonds of Cationic and Neutral mMoDTC^a^

	length (Å)			size (deg)	
bond	cationic	neutral	angle	cationic	neutral
H(1)–C(2)	1.10	1.10	C(2)–N(3)–C(2′)	115	116
C(2)–N(3)	1.46	1.46	C(2)–N(3)–C(4)	123	122
N(3)–C(4)	1.32	1.34	N(3)–C(4)–S(5)	123	124
C(4)–S(5)	1.73	1.73	S(5)–C(4)–S(5′)	113	111
S(5)–Mo(6)	2.44	2.44	C(4)–S(5)–Mo(6)	87	89
Mo(6)–O(7)	1.69	1.70	S(5)–Mo(6)–O(7)	111	109

a The numbers in brackets follow the numbering proposed in Figure 1.

### Fragmentation of Isolated mMoDTC

**Figure 7:**
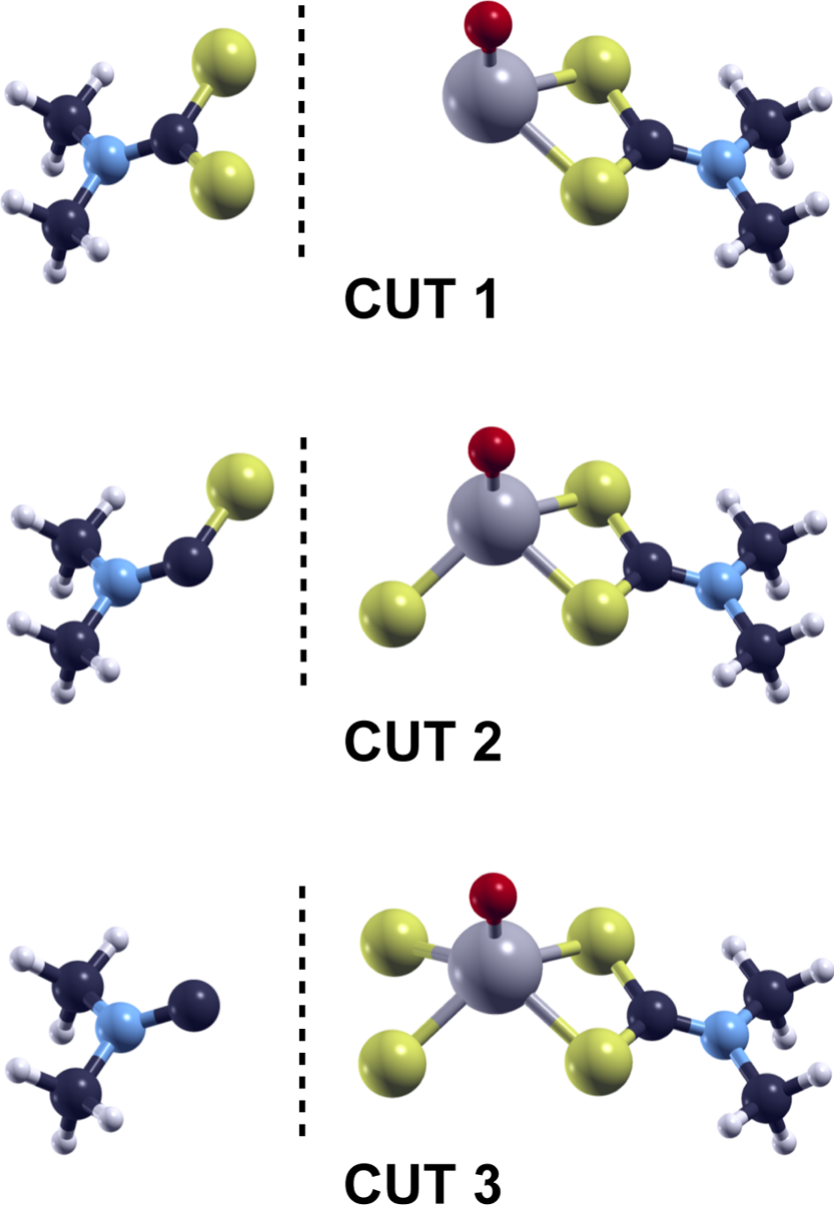
Schematic representation of the dissociation patterns of mMoDTC.

We estimated the bond strengths in mMoDTC in order to understand which is the most probable dissociative pattern. In analogy with our previous publication,^12^ we calculated the fragmentation energies Δ*E*_frag_ in the following way
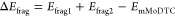
1where *E*_frag1_, *E*_frag2_, and *E*_mMoDTC_ are the total energies of the two molecular fragments originating by the dissociation of mMoDTC and the whole complex, respectively. The geometry of the fragments was kept frozen to prevent any spatial rearrangement of the atoms that could influence the energy values. The atoms with a broken bond were not passivated by other species, and spin polarization was considered to allow the electronic configurations to reach spin multiplicities higher than singlets. We took into account three different ways of dividing the complex: cut 1 corresponds to the homolytic breaking of the two S–Mo bonds, while cut 3 corresponds to the breaking of the two C–S bonds, proposed in the dissociative mechanisms by Grossiord et al.^5^ and by Khaemba et al.,^6^ respectively. Cut 2 is the intermediate pattern in which one S–Mo bond and one C–S bond are broken. A schematic representation of these fragmentation patterns is presented in Figure 7. The electronic configuration of the fragments of the cationic form of mMoDTC was left free to reach the minimum energy state, which corresponds to the lowest spin multiplicity (doublets). The absolute values of fragmentation energies are summarized in Table 3.

**Table 3:** Fragmentation Energies of Isolated mMoDTC and sMoDTC, Expressed in eV^a^

	cationic mMoDTC	neutral mMoDTC	sMoDTC
cut 1	4.8	4.8	4.1
cut 2	5.3	4.0	4.6
cut 3	7.1	5.5	6.7

a In the first column and the second column, energy values are reported for the cationic and neutral forms of mMoDTC, respectively, while in the last column, the energy values for the binuclear structure sMoDTC are taken from ref (12).

The calculations revealed that the most favorable destination for the positive charge after the dissociation is the central metallic unit. Cut 1 turned out to be the most favorable dissociation pattern, as in the case of the dimeric structure, as it required 0.5 and 2.3 eV less than cut 2 and cut 3, respectively. By carrying out the same investigation on the neutral form of mMoDTC, we found out that describing the molecular fragments as doublets was insufficient to estimate the strength of the broken bonds. In fact, cut 2 appears to be the most favorable fragmentation for the neutral compound. Cut 2 corresponds to an intermediate situation between cut 1 and cut 3, and its fragmentation energy should also lie between the energies of the other two patterns, according to the results of previous fragmentation analyses carried out for sMoDTC and Ni(acac)_2_.^12,34^ Indeed, considering the Mo-containing fragment obtained by cut 2 as a doublet leaves the unpaired electron on a sulfur atom, which is more electronegative than molybdenum. This may explain the lower fragmentation energy of cut 2. The unpaired electron, indeed, remains on the Mo atom in the other fragmentation patterns. For a detailed explanation of this aspect, the reader is referred to the Supporting Information, where a schematic representation of these fragments is shown. A more appropriate way of estimating the strength of the bonds in mMoDTC would be to consider the fragments as quartets, which better describe the electronic configuration obtained by the homolytic dissociation of the complex. Low spin multiplicities can be obtained after a relaxation of the electronic configuration of the fragments, after which two electrons originally involved in two different bonds turn into an electron pair on the same orbital. This process minimizes the energy of the system. However, two molecular fragments with only one unpaired electron each cannot recombine into a whole mMoDTC molecule unless the electron pairs are separated again. Therefore, we compared the fragmentation energies of neutral mMoDTC by considering the spin multiplicities of doublets and quartets with different approaches. The first of the three columns for each spin multiplicity in Table 4 reports the fragmentation energies calculated by Quantum ESPRESSO by using the PBE^35^ approximation, while the second and third columns report the same values calculated with Gaussian 09 using the PBE and the PBE0^35^ approximations, respectively. While PBE0 in Gaussian is able to reproduce the same trend seen with Quantum ESPRESSO, it turns out that the correct energy trends are recovered when the fragments are considered as quartets because at least one unpaired electron is localized on the Mo atom for all the fragmentation patterns. By considering a high spin multiplicity, the most favorable fragmentation of neutral mMoDTC is confirmed to be cut 1, in analogy with the MoDTC dimer.

**Table 4:** Fragmentation Energies of Neutral mMoDTC, Expressed in eV, Calculated with Different Techniques and Compared by Different Spin Multiplicities of the Fragments

	doublets			quartets		
	PBE (QE)	PBE (G09)	PBE0 (G09)	PBE (QE)	PBE (G09)	PBE0 (G09)
cut 1	4.8	5.5	5.5	8.1	8.5	8.5
cut 2	4.0	4.4	4.0	8.6	8.7	8.8
cut 3	5.5	6.1	5.6	9.6	9.8	9.7

### Chemisorption and Fragmentation on Iron

**Figure 8:**
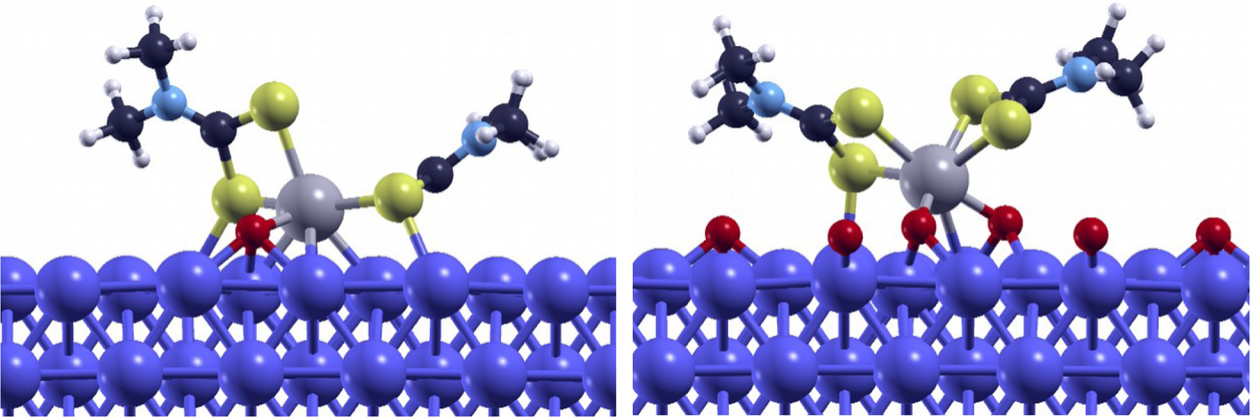
Optimized configuration for mMoDTC adsorbed on the clean (left) and oxygen-passivated (right) iron (110) surfaces.

Figure 8 shows the optimized configuration for the adsorption of mMoDTC on the clean and passivated Fe(110) surfaces. These orientations of the complexes on the substrates might not be the absolute minimum configurations, yet they offer a clear qualitative description of the differences in the adsorption on the clean and the passivated surfaces. The passivation of iron is achieved by 0.25 monolayers of oxygen atoms put in the threefold coordination site of the metal as it is the most favorable configuration for this element on iron.^36^ Both the cationic and the neutral forms of mMoDTC are chemically bound to the surface through the central unit. The passivation weakens the chemisorption as less atoms of the complex are able to bind to the free sites of iron. This is reflected upon the adsorption energies of the complex, calculated as
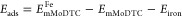
2where *E*_mMoDTC_^Fe^ and *E*_iron_ are the total energies of the chemisorbed complex on the substrate and of the iron slab alone, respectively. The adsorption energies of cationic mMoDTC turned out to be −11.4 and −9.7 eV in the case of the clean surface and the passivated surface, respectively. Such favorable values of energy for the chemisorption are due to the stabilization effect of the positive charge of the complex interacting with the metallic substrate. In particular, a Bader charge analysis^37−40^ revealed that the positive charge is completely redistributed only on the topmost iron layer, which is the closest to the complex, with almost no effect on the underlying layers. The absence of the positive charge, in fact, leads to adsorption energies of −3.2 and −1.4 eV for the clean and passivated surfaces, respectively.

We additionally verified what is the most favorable fragmentation pattern for the complexes adsorbed on iron. For this purpose, we optimized the geometry of the fragments of mMoDTC on iron, in analogy to the investigation carried out for the dimeric complex,^30^ and we calculated the dissociation energies as
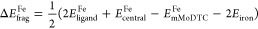
3where *E*_frag1_^Fe^ and *E*_frag2_^Fe^ are the total energies of the lateral ligands and the central units of mMoDTC adsorbed on iron, respectively. Cuts 1 and 3 were the dissociative patterns considered to generate the molecular fragments adsorbed on iron. The most favorable fragmentation for the complex on the substrate turned out to be Cut 3 with a favorable fragmentation energy of −2.2 eV, compared to an unfavorable value of 0.2 eV for cut 1. This result is in contrast with the case of the isolated compound. We observed that this phenomenon also occurs for the dimeric MoDTC structures and complexes with a similar structure.^30,34^ This phenomenon is due to the capability of the metallic substrate to stabilize more efficiently the fragments originating by separating the complexes between the chalcogen atoms and the carbon atom of the ligand unit. mMoDTC compounds follow the general rules derived for these small organometallic complexes

- While the fragmentation of isolated complexes is not favorable, the metallic substrate is able to stabilize the molecular fragments and the dissociation energies become favorable.
- The complexes on the metallic substrates can follow alternative dissociative patterns, in which the weakest bonds are not the first ones to be broken. It is more probable to break the molecules between the carbon atom and the chalcogen atoms of the ligand unit.

**Figure 9:**
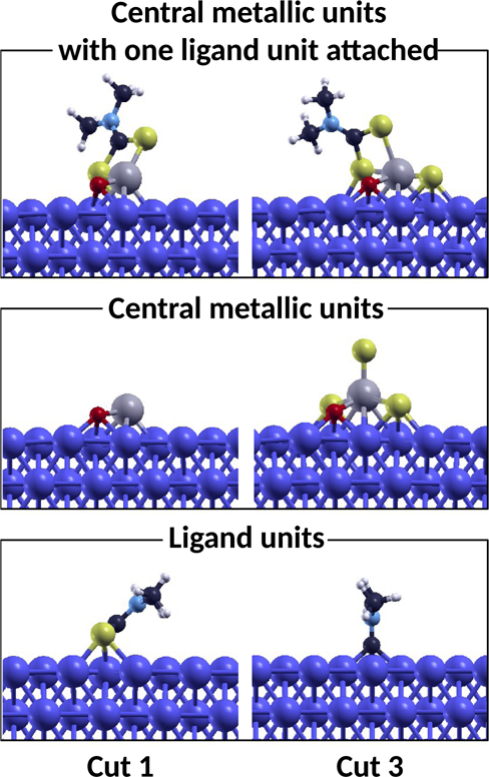
Fragments of mMoDTC originating by each individual step of the dissociation of the ligand units: central metallic units with one ligand unit attached and central metallic units alone and ligand units alone obtained from cut 1 and 3, respectively.

The fragmentation energies obtained from eq 3 actually correspond to the average of the two values corresponding to the dissociation of the two ligand units, one after the other. A more precise estimation would require to calculate the dissociation energies for each step of the reaction. We carried out these calculations for both cationic and neutral mMoDTC, with the results summarized in Table 5. Figure 9 shows all the fragments originating by the individual steps of the dissociation. An additional adsorption configuration could be possible for the molecular fragments on iron compared to the ones presented in the figure. However, the complete screening of all the possible adsorption geometries of these fragments goes beyond the purpose of this work. These adsorption configurations were chosen to merely show that the dissociation mechanism of these compounds on iron is a complex multi-step reaction. It turns out that the first dissociative step is always more favorable than the second dissociation step. By comparing the dissociation energies of mMoDTC and sMoDTC, the values for cut 3 are essentially similar, while for cut 1, mMoDTC is much harder to break. Moreover, the dissociation energies of the neutral compound are generally lower and hence more favorable than the ones of the cation. This is due to the strong chemisorption of the cationic compound induced by the strong reactivity of the metallic core hosting a radical electron. Such an electronic configuration makes the mMoDTC cation stickier than the neutral compound, and the stronger binding between the complex and the substrate can hinder the subsequent dissociative steps which can be useful to form the desired MoS_2_ clusters. These features can be an explanation for the behavior observed in the tribological tests. In fact, the fraction which is the richest in mMoDTC presents slower kinetics in the activation to form MoS_2_. An unbalance between monomeric and dimeric structures of MoDTC in oil mixtures limits the friction reduction performances of the lubricants as MoS_2_ needs to be generated continuously during sliding.

**Table 5:** Fragmentation Energies of Cationic and Neutral mMoDTC, Expressed in eV, for Each Individual Step of the Dissociation

	cationic		neutral	
	cut 1	cut 3	cut 1	cut 3
first detachment	–0.1	–3.0	–0.4	–3.4
second detachment	0.4	–1.4	–0.1	–1.7
total	0.3	–4.3	–0.5	–5.0

**Figure 10:**
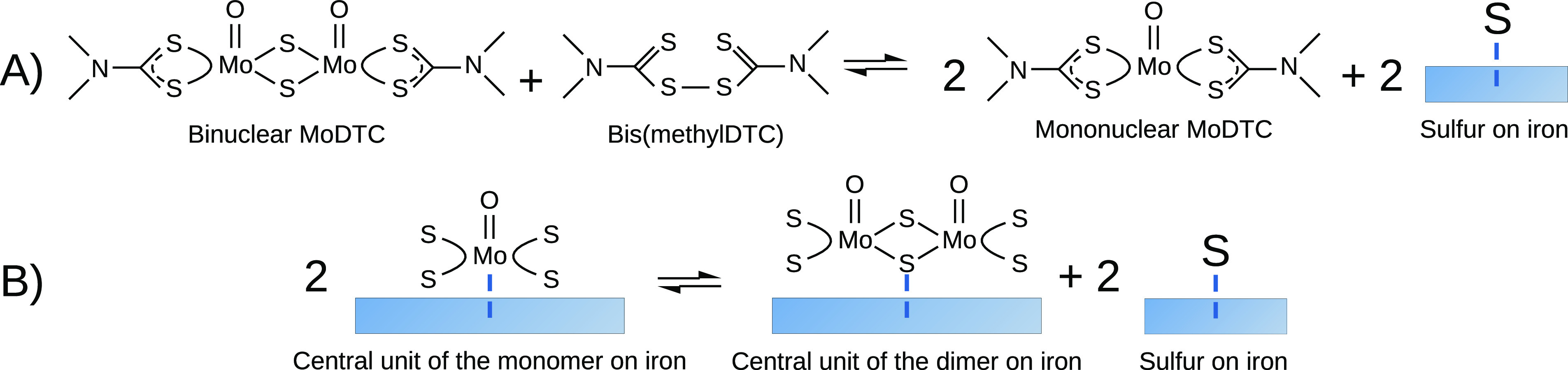
Scheme of two hypothetical reactions where the mononuclear and binuclear MoDTC molecules in the mixture (A) or their central units on iron (B) are in equilibrium.

Finally, we considered two model reactions that aim at better understanding the equilibrium between monomeric and dimeric forms of MoDTC. These reactions are schematically represented in Figure 10. In reaction A, we consider a transformation of the binuclear MoDTC structure into two mononuclear structures when an excess of ligand units is present in the form of bis-DTCs. This hypothetical reaction is supposed to occur in the mixture in the proximity of a metallic substrate able to host the sulfur atoms in excess. The energy exchanged in this reaction can be written as
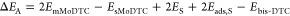
4where *E*_sMoDTC_, *E*_S_, and *E*_bis-DTC_ are the total energies of the binuclear MoDTC structure, an isolated sulfur atom, and the bis-DTC molecule, respectively, and *E*_ads,S_ is the adsorption energy of a sulfur atom on the Fe(110) surface. The reaction energy Δ*E*_A_ turned out to be −4.31 eV, indicating that the conversion of a binuclear MoDTC molecule into two mononuclear molecules in the mixtures is favorable in the presence of bis-DTCs and a suitable destination for the sulfur atoms in excess. In order to better understand whether the central units of mMoDTC adsorbed on iron are more stable than the central units of sMoDTC under the same conditions, we considered a different chemical reaction where two central units of mMoDTC on iron combine into one central unit of sMoDTC and release two sulfur atoms on the iron surface. The energy exchanged in this reaction can be written as
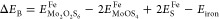
5where , , and *E*_S_^Fe^ are the total energies of the central units of sMoDTC and mMoDTC and of an S atom adsorbed on iron, respectively. In this case, the reaction energy Δ*E*_B_ turned out to be −0.53 eV, implying that a single central unit of the binuclear compound and two sulfur atoms adsorbed on iron are more stable than two central units of the mononuclear compound. This suggests that central units obtained from mMoDTC could recombine into binuclear cores on the iron substrates, becoming more similar to MoS_2_ in terms of stoichiometry, in combination with the release of two S atoms. After providing enough energy in the system to cause the dissociation of mMoDTC, the central units of the complex near each other have the possibility to assemble into wider structures, which is an advantage for the formation of MoS_2_. On the other hand, starting to build a MoS_2_ tribolayer from the dimeric MoDTC structures can be more beneficial as the central units of the dimers require one recombination step less than the monomers.

## Conclusions

In this work, we described the properties of one of the components of lubricant mixtures containing MoDTC: the mononuclear structure referred to as mMoDTC. Mononuclear structures are, in fact, a group of MoDTC compounds present in the lubricants along with the binuclear compounds. For the first time, we were able to separate chemically different fractions from the MoDTC-containing lubricant S525 and to characterize such fractions by means of XRF, mass spectrometry, EPR, UV/vis spectroscopy, and tribology tests. These fractions turned out to be different in terms of composition, elemental concentration of molybdenum and sulfur, electronic absorption, and tribological performance. In particular, fractions containing a larger amount of mMoDTC showcase a green color instead of yellow, and they present slower kinetics in the formation of the MoS_2_ tribolayers. To shed light on these aspects, we calculated the electronic and structural properties of mMoDTC, which are similar to the ones of the dimeric structures. However, the lower ionization energy and fundamental gap make the monomer a reactive compound that can quickly interact with other chemical species present in the mixtures. A consequence of this enhanced reactivity is that the chemisorption of the cationic compound to the metallic substrate is stronger than that in the case of the dimer. This is reflected upon the higher dissociation energies of cut 1 compared to the corresponding values of the dimer, while the dissociation energies of cut 3 are very similar between the two complexes. On the other hand, the neutral form of mMoDTC chemisorbs more weakly than the dimer, indicating that the enhanced reactivity can be observed only after ionization.

The fact that cationic mMoDTC can easily interact with other chemical species through the unpaired electron could be seen as a drawback for the lubricant oil as undesired chemical species can be formed under tribological conditions, slowing down the kinetics to form MoS_2_. However, some features of the mononuclear compound are still beneficial in the friction performance of the lubricant mixture, namely, the possibility to donate easily one electron from mMoDTC to the metallic substrate and to generate a strong interaction between the molecule and iron, its dissociation mechanism, which is essentially the same compared to the one of sMoDTC, and the fact that the central core units obtained after dissociation can recombine into larger units. The combination of experimental and computational investigations showed that only a good balance between the different MoDTC structures ensures the low-friction performances of the lubricant because the mononuclear and binuclear compounds have different roles to play during sliding.

## Supporting Information Available

Concentration of sulfur and molybdenum in the collected fractions; electronic spectra of fractions 7, 8, and 9; comparison of the orbitals of neutral and cationic mMoDTC; and details on the electronic structure of the molecular fragments (PDF)
