# Supplementary material for: Experimental and Ab Initio Characterization of Mononuclear Molybdenum Dithiocarbamates in Lubricant Mixtures
Source: Langmuir. 2021 Apr 13;37(16):4836–46. doi: 10.1021/acs.langmuir.1c00029 (PMC8154871; doi:10.1021/acs.langmuir.1c00029)
Supplement: Supplementary file 1 — la1c00029_si_001.pdf [file la1c00029_si_001.pdf]

Supporting Information: Experimental and Ab-Initio Characterization of Mononuclear Molybdenum Dithiocarbamates in Lubricant Mixtures

Gabriele Losi,[†,§] Stefan Peeters,[†,§] Franck Delayens,[‡] Hervé Vezin,[¶] Sophie Loehlé,[‡] Benoit Thiebaut,[‡] and Maria Clelia Righi[*,†]

†*Department of Physics and Astronomy, University of Bologna, 40127 Bologna, Italy*

‡*Total Marketing and Services, Chemin du Canal BP 22, 69360 Solaize, France*

¶*University of Lille, CNRS, UMR 8516 - LASIRE - Laboratoire Avancé de Spectroscopie pour les Interactions, la Réactivité et l'Environnement, F-59000 Lille, France*

§*Department of Physics, Informatics and Mathematics, University of Modena and Reggio Emilia, I-41125 Modena, Italy*

E-mail: clelia.righi@unibo.it

Concentration of sulfur and molybdenum in the collected fractions

Table S1 reports the concentrations of sulfur and molybdenum in most of the collected fractions obtained by the separation of S525. Fractions 7, 8 and 9 are the most concentrated in terms of product, as they showcase the highest sulfur and molybdenum concentration. The values of Mo/S ratio reported in Table S1 can be compared with the ideal Mo/S ratio in individual MoDTC structures shown in Table S2.

Table S1: Concentrations of sulfur and molybdenum in the collected fractions.

Fraction	S (ppm)	Mo (ppm)	Mo/S ratio %
5	0.0	<5	N/A
6	32.9	26.8	81.4
7	456.3	392.6	86.0
8	415.5	402.2	96.8
9	434.3	432.0	99.5
10	32.9	31.4	95.4
11	20.4	15.6	76.5
17	42.8	29.7	69.4

Table S2: Mo/S mass ratio in individual MoDTC compounds. In the columns labelled as "#O-#S", the first (second) number corresponds to the number of O (S) atoms in the chemical structures.

Mononuclear (1 Mo)		Binuclear (2 Mo)	
#O-#S	Mo/S ratio %	#O-#S	Mo/S ratio %
0-5	59.8	0-8	74.8
1-4	74.8	1-7	85.5
2-3	99.7	2-6	99.7
3-2	149.6	3-5	119.7
4-1	299.2	4-4	149.6
		5-3	199.4
		6-2	299.2
		7-1	598.5

Electronic spectra

Figure S1 shows the electronic spectra of Fractions 7, 8 and 9. In the UV range, the differences between the fractions are small, although still detectable, while an increased absorption can be detected between 500-700 nm for Fraction 9, justifying the lack of the green color observed in the other samples.

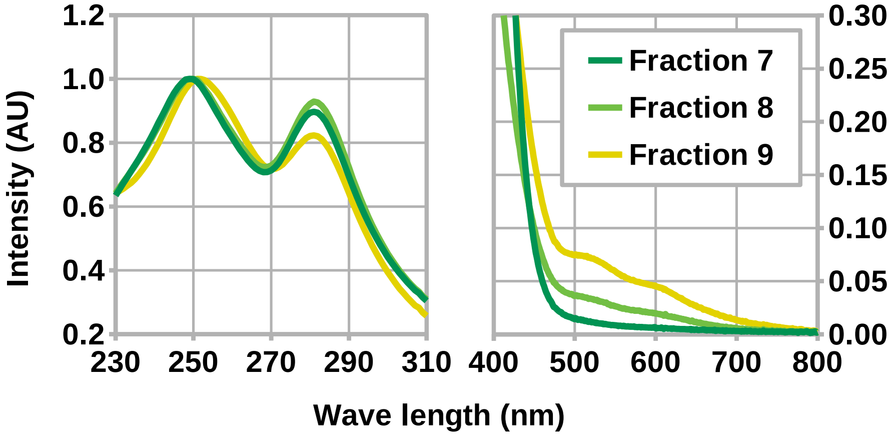

Figure S1: UV (left) and visible (right) spectra of Fractions 7, 8 and 9. The intensity of the UV peaks at 250 nm was normalized, while the visible spectra were not scaled.

Orbitals of neutral and cationic mMoDTC

In Figure S2, the calculated orbitals of the neutral and cationic forms of mononuclear MoDTC are reported for comparison. While the populated orbitals are essentially identical, the calculated LUMOs are quite different, due to the difficulty to accurately describe empty orbitals within density functional theory.

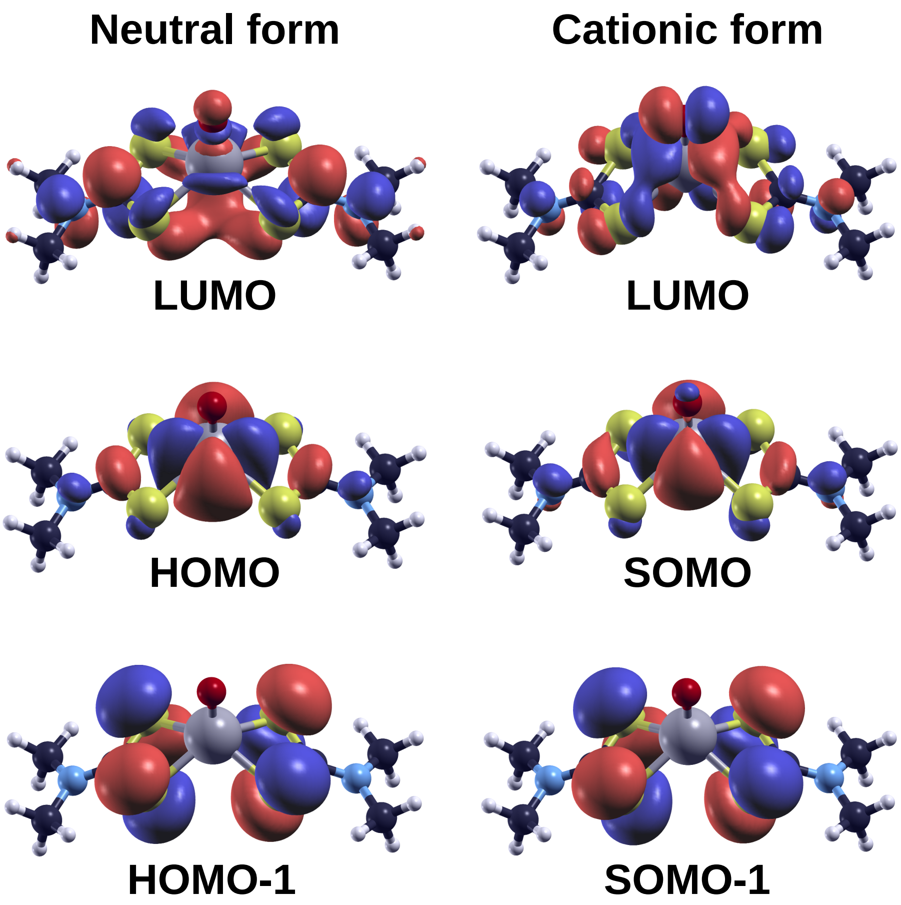

Figure S2: Frontier molecular orbitals of the neutral and cationic forms of mMoDTC. The isovalue for all the plots is 0.002. The red and blue colors of the isosurfaces represent the positive and negative sign, respectively, of the electronic wave function.

Electronic structures of the fragments of isolated mMoDTC

Cut 2 appeared as the most favorable fragmentation pattern for the neutral compound. This pattern, in which one sulfur atom remains attached to the Mo atom, while the other sulfur atoms is bound to the C atom of the ligand unit, is an intermediate situation between Cut 1 and Cut 3. Our fragmentation analysis aims at estimating which are the weakest bonds to break. Therefore, we would expect Cut 2 to provide a fragmentation energy which is intermediate between the energies of Cut 1 and Cut 3. This would be the case if we were able to separate the two fragments quickly enough to avoid any relaxation of the electronic configuration. This schematic view of the dissociation is impossible to achieve experimentally because the electronic motion is much faster than the ionic motion. Furthermore, all the calculated fragmentation energies are positive, meaning that at zero temperature a whole molecule is more stable than its separated fragments. However, this approach can be useful to estimate the strength of the interaction between atoms involved in chemical bonds.

During the self-consistent calculations, the code optimizes the electronic structure to reach the minimum energy state. Without any constraints, the lowest energy is obtained with the lowest spin for all the fragments. By considering Cut 2, the fragment containing the Mo atom as a doublet has an unpaired electron localized on the sulfur atom with a broken bond, as shown in Figure S3. The equivalent fragments obtained from Cuts 1 and 3, instead, are characterized by having one unpaired electron on the Mo atom. In particular, in the fragment obtained by Cut 1 the Mo atom lacks two S atoms on its left side and the other ligands account only for three bonds, leaving three valence electrons on molybdenum. Out of three electrons, two become an electron pair in the $5s$ orbital, while the third one remains unpaired in a $4d$ orbital. Similarly, only one unpaired electron is localized on the Mo atom of the fragment from Cut 3, because a bond is formed between the sulfur atoms that would be otherwise bound to carbon.

Sulfur is more electronegative than molybdenum, so an unpaired electron is more stabilized on the S atom rather than on the Mo atom. This is most probably the reason why Cut

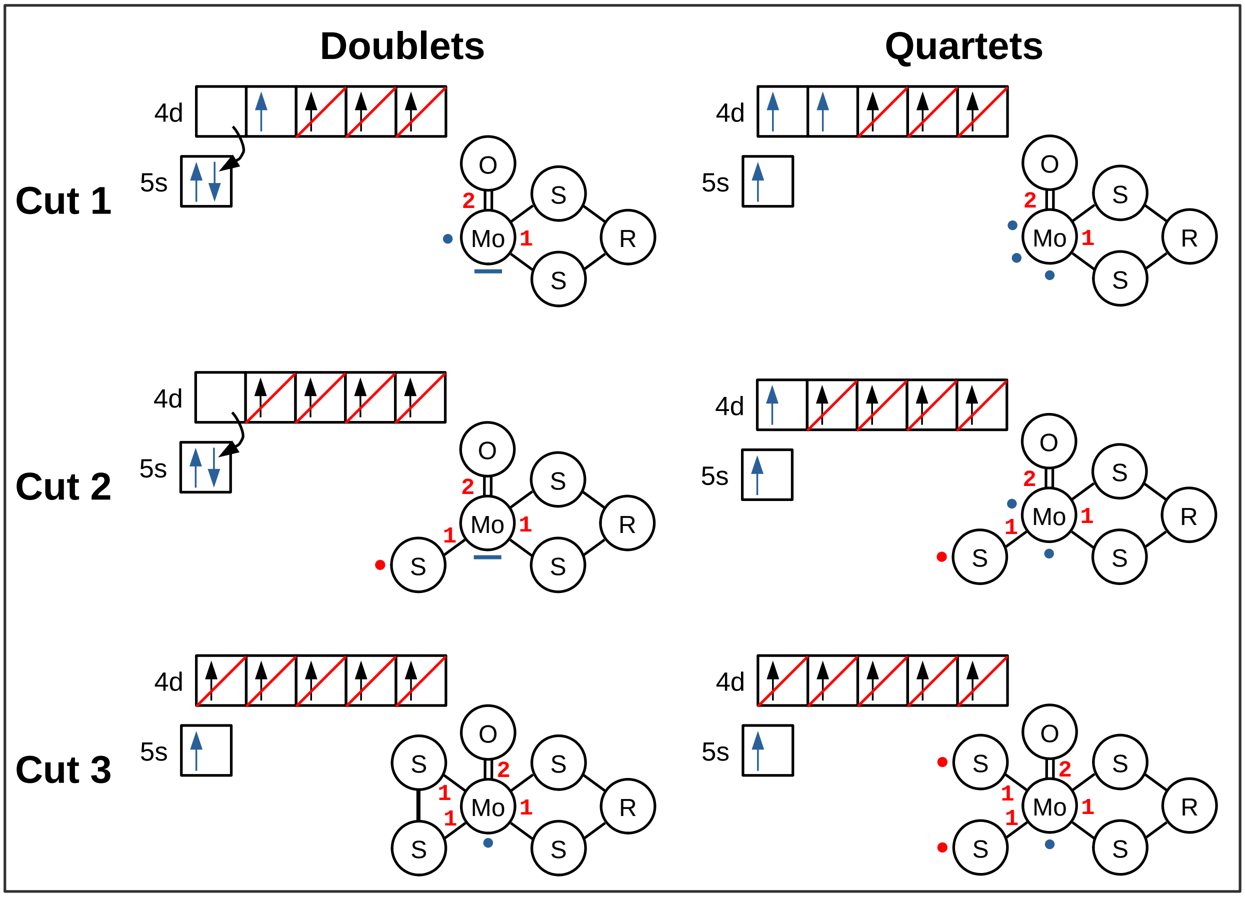

Figure S3: Largest fragments obtained by the dissociation of mMoDTC, following different fragmentation patterns and considering the spin multiplicities of doublets and quartets. For each fragment, the electronic configuration of the molybdenum atom and a simplified chemical structure are reported. The red numbers surrounding the Mo atoms indicate the order of the respective bonds.

2 has a lower fragmentation energy than Cut 1 and Cut 3, when considering low spin multiplicities. However, such fragments cannot be used to build back mMoDTC. Let us consider, for example, the left and right fragments obtained by Cut 2. When these fragments are doublets, one unpaired electron is localized on the S atom of the ligand unit while the other unpaired electron is on the S atom attached to the molybdenum atom. By putting these two radicals together, one would obtain an organomolybdenum complex connected to a tertiary amine through a disulfide bridge, rather than a molybdenum dithiocarbamate. Considering the fragments as quartets is a better way to estimate the strength of the bonds, because a recombination of such fragments would restore the original mMoDTC structure.

It is energetically expensive to reach high spin multiplicities, as indicated by the calculated fragmentation energies. Yet, a dissociation of these compounds in the gas phase is very unlikely to occur, as previously explained. Therefore, the purpose of this fragmentation analysis is not to suggest a possible dissociation mechanism, but rather to qualitatively compare the energy costs to break selected bonds.
